# Genome sequence of *Babesia gibsoni* detected in a domestic dog in New Zealand

**DOI:** 10.1128/mra.01083-25

**Published:** 2025-11-18

**Authors:** Andrew Wilson, Ruy Jauregui, Rudolfo Bueno, Douglas Begg, Michaela Hannah, Elisha Sheeba, Mark Bestbier, Daniel Green, Joseph O'Keefe, Michelle McCulley

**Affiliations:** 1 Animal Health Laboratory, Diagnostics, Readiness and Surveillance, Biosecurity New Zealand, Ministry for Primary Industries, Upper Hutt, New Zealand; Rochester Institute of Technology, Rochester, New York, USA

**Keywords:** *Babesia*, gibsoni, New Zealand

## Abstract

We report the draft genome sequence of *Babesia gibsoni* detected in a domestic dog in New Zealand. A high-quality near-complete genome, comprised of an apicoplast, mitochondrion, and four chromosomes, was produced through long-read metagenomic sequencing.

## ANNOUNCEMENT

*Babesia gibsoni* (*B. gibsoni*) is a protozoan parasite belonging to the family *Babesiidae* of the order *Piroplasmida*. Commonly transmitted through the bite of infected ticks, *B. gibsoni* invades the erythrocytes of an infected host and can cause babesiosis, an illness characterized by clinical symptoms such as fever, anemia, and lethargy (1), with severe cases leading to organ failure and death (2). *B. gibsoni* is globally widespread but had not previously been detected in New Zealand.

In 2024, blood smears taken from a domestic dog (exposed to an imported dog) presenting with anemia highlighted suspicious inclusions that were consistent with babesiosis. A peripheral blood sample, collected into an EDTA vacutainer, was delivered to the New Zealand Ministry for Primary Industries Animal Health Laboratory for exotic disease investigation. DNA was extracted using the QIAamp DNA Mini kit (Qiagen). Using a nested PCR to detect and differentiate between canine piroplasm species, the sample produced an approximately 800-bp product after amplification (3). Amplicons were electrophoresed and visualized, followed by excision of the PCR product from the agarose gel. The excised product was then purified using the ExoSAP-IT PCR product cleanup reagent (Thermo Fisher Scientific).

The PCR product was prepared for Sanger sequencing using the BigDye Terminator v3.1 Cycle Sequencing Kit (Thermo Fisher Scientific) followed by reaction cleanup using the BigDye XTerminator Purification Kit (Thermo Fisher Scientific). Purified products were loaded onto a SeqStudio Genetic Analyzer (Applied Biosystems) for Sanger sequencing. After sequencing, forward and reverse primer sequences were *de novo* assembled in Geneious Prime v2021.1.1 (https://geneious.com). The resulting consensus sequence, approximately 750 bp of contiguous read length, was interrogated using NCBI BLAST. The top BLAST result was *B. gibsoni*, aligning with 99.9% pairwise ID (GenBank ID: OQ727057). Whole genome sequencing using a long-read metagenomic approach was then performed using the original DNA extract.

Libraries for long-read metagenomics were prepared using the Rapid Barcoding Kit V14 24 (SQK-RBK114.24, Oxford Nanopore Technologies [ONT]) and sequenced on a MinION Mk1B with a R10.4.1 flow cell (ONT). Basecalling was performed with Dorado v7.4.14 (ONT) using the high-accuracy model v4.3.0. Raw reads were trimmed using BBDuk v38.84 (4) and then mapped to a reference *B. gibsoni* genome (GenBank IDs: CP141525–CP141530) using Minimap2 v2.24 (5) to generate a consensus-based assembly. In total, six scaffolds were generated, representing the apicoplast, mitochondrion, and four chromosomes. Genome completeness was estimated with BUSCO v5.8.2 using the apicomplexa_odb12 data set (6). Genome assembly statistics are presented in Table 1. Terminal ends of each chromosome were poorly represented in each assembled chromosomal sequence; therefore, genome ends could not be established. The mitochondrial sequence reported here was characterized as circular based on the reported circularity of the reference sequence.

**TABLE 1:** Genome assembly statistics for the reported *B. gibsoni* genome

Feature or genome segment	Assembly statistic or segment size	Number of reads mapped	Depth of coverage
Features			
Raw read number	555,825		
Number of mapped reads	21,539 (3.9%)		
Raw read N50	11.88 kb		
Median Q-score of raw reads	>Q15		
Total genome size	7.93 Mb		
Total GC content	44.0%		
Number of scaffolds	6		
Scaffold N50	2 Mb		
Percent gaps	0.10%		
Completeness	98.1%		
Genome segments			
Apicoplast	28.4 kb	384	87×
Mitochondrion	5.87 kb	165	63×
Chromosome 1	0.69 Mb	2,017	16×
Chromosome 2	2.10 Mb	4,331	12×
Chromosome 3	2.76 Mb	9,580	22×
Chromosome 4	2.38 Mb	5,062	12×

All laboratory protocols detailed above were performed according to the manufacturer’s instructions. For software, default parameters were used.

In summary, we present a high-quality, near-complete draft genome of *B. gibsoni* detected in a domestic dog in New Zealand. The genome was sequenced and characterized using long-read metagenomic approaches, highlighting the critical role of genomics in enabling rapid biosecurity responses.

## Data Availability

Raw sequence data have been submitted to NCBI’s Sequence Read Archive (SRA) under the BioProject accession number PRJNA1329508. This Whole Genome Shotgun project has been deposited at DDBJ/ENA/GenBank under the accession number JBRECT000000000. The version described in this paper is version JBRECT010000000.
